# The Role of Analytical Flexibility in Determining Mental Health Biomarkers

**DOI:** 10.1016/j.bpsgos.2022.07.008

**Published:** 2022-10-14

**Authors:** Janine Bijsterbosch

**Affiliations:** Department of Radiology, Washington University School of Medicine, St. Louis, Missouri

SEE CORRESPONDING ARTICLE ON PAGE 489

Many studies have reported alterations in functional brain organization and connectivity associated with mental health disorders such as anxiety and depression, also known as disorders of anxious misery. However, clinical translation of this work into robust magnetic resonance imaging–based mental health biomarkers has remained elusive. There are several challenges that contribute to this lack of clinical translation, including limited reproducibility of findings (1) and analytical flexibility (2) in terms of both brain representations (3) and brain-symptom modeling approaches (4). In the current issue of *Biological Psychiatry: Global Open Science*, Seok *et al.* (5) assessed the impact of analytical flexibility by testing different brain-symptom modeling approaches to determine which model best predicts mental health symptom dimensions. Their findings provide important insights into the multivariate brain basis of mental health and the challenges and opportunities that arise from analytical flexibility.

**Figure 1:**
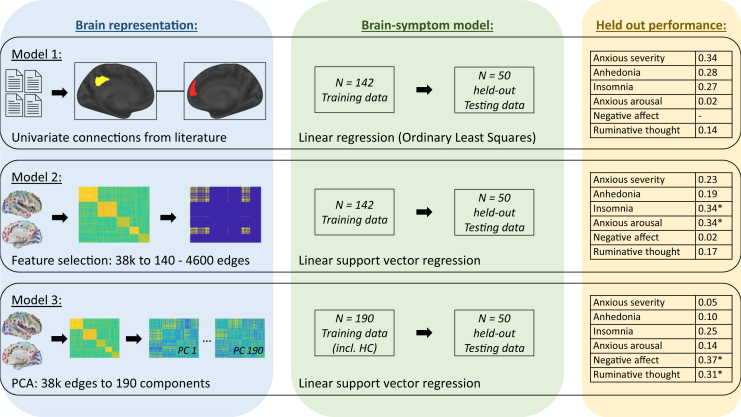
Seok *et al.* (5) evaluated analytical flexibility by testing 3 different brain-symptom models across 6 symptom dimensions. (Top panel) Model 1 was a literature-based model involving univariate linear regressions between symptom scores and connectivity between selected pairs of brain regions that were informed from the published literature. (Middle panel) Model 2 was a multivariate model involving feature selection out of a parcellated 278 × 278 connectivity matrix followed by support vector regression. In model 2, the number of features to retain was optimized, resulting in 140 to 4600 features across symptom dimensions. (Bottom panel) Model 3 was a multivariate model involving principal component analysis (PCA) of the same 278 × 278 connectivity matrix followed by support vector regression. Training data for model 3 included healthy control (HC) participants that were not used in models 1 and 2. Held-out performance in the yellow column summarizes Pearson correlations between actual and predicted symptom dimensions in the held-out testing data (*n* = 50) as reported in Table 3 in Seok *et al.* (5). Asterisks indicate the models that both performed best and reached multiple-comparisons corrected significance.

Seok *et al.* (5) started by identifying 6 data-driven mental health symptom dimensions of anxiety sensitivity, anxious arousal, ruminative thought, anhedonia, insomnia, and negative affect using hierarchical clustering. Subsequently, the authors compared 3 different brain-symptom modeling approaches to identify associations between symptom dimensions and functional connectivity measures, including 1 univariate approach and 2 different multivariate approaches (Figure 1). Testing in a modest held-out sample revealed that different symptom dimensions were best predicted using different multivariate brain-symptom modeling approaches (Figure 1). As such, Seok *et al.* (5) used a data-driven, symptom-level framework inspired by the Research Domain Criteria to identify latent symptom dimensions of mental health and to systematically compare brain-symptom modeling approaches.

Seok *et al.*’s (5) findings emphasize the highly multivariate nature of functional connectivity correlates of mental health symptomatology—findings further supported by other recent work (6,7). Although some univariate results from model 1 were replicable in the held-out sample (Figure 1), these did not survive the strict multiple comparisons corrections necessitated when performing a finite set of independent univariate tests. Conversely, multivariate modeling approaches leverage correlations across many connections to identify brain patterns linked to symptom dimensions, thereby mitigating the strict multiple comparisons correction requirements because statistical inference is performed at the level of brain patterns rather than individual connections. The use of multivariate data-driven models to predict mental health symptomatology holds great promise, especially toward the goal of developing a more personalized framework for diagnosis and treatment in psychiatry. Nevertheless, the complexity of multivariate brain patterns may hinder the interpretability of findings, as they point to a highly multifaceted neurobiological etiology of mental health.

The article by Seok *et al.* (5) is among the first to evaluate the role of analytical flexibility in brain-symptom modeling approaches [see also (4)], echoing previous work in psychology (8) and task-based functional magnetic resonance imaging (9). The results raise important advantages of systematically evaluating analytical flexibility. For example, good prediction accuracy was achieved by only 1 of the 3 modeling approaches for some symptom dimensions such as negative affect, which may have been missed or reported as negative findings had the authors used only a single modeling approach. Other symptom dimensions such as insomnia were relatively well predicted using multiple approaches, and post hoc exploration of the weights revealed convergence across the models in terms of which connections contributed to prediction results. Hence, systematically testing analytical flexibility is critically important to gain insights into which analytical decisions have the greatest impact, how findings map or converge across different approaches, and which approaches are best suited for which research questions. To achieve these benefits, it is important to report results for all approaches that were tested to avoid problematic aspects of researcher degrees of freedom and to perform appropriate statistical corrections across all tests (8). Beyond the study by Seok *et al.* (5), future studies will likely benefit from even broader evaluations of analytical flexibility by incorporating additional modeling approaches and evaluating different brain representations (3).

Compared with previous work, Seok *et al.* (5) used a relatively larger sample size (*n* = 142 training) and added the use of a held-out testing sample (*n* = 50 testing). However, recent work has highlighted the importance of even larger samples to avoid sampling variability, due to the small effect size of most brain-symptom associations (10), suggesting that future work should aim to adopt even larger samples. Furthermore, the use of k-fold cross-validation and bootstrapping instead of a single held-out test group may be beneficial to quantify variance in prediction performance across many iterations of held-out samples (4). The study by Seok *et al.* (5) also highlights the challenge in conceptualizing and interpreting analytical differences across modeling approaches. Model 2 is interpreted as using “subsets of the connectome,” whereas model 3 is conceptualized as a “broad representation of the entire connectome” (Figure 1). Although these interpretations are not inaccurate, it should be noted that both models start from the full connectome and both models involve dimensionality reduction through either feature selection, in the case of model 2, or principal component analysis, in the case of model 3. Therefore, it is challenging to meaningfully interpret and conceptualize such analytical differences, especially given the complexities of interactions between the definition of features, the optimization function of the prediction algorithm, the sample size, etc.

In conclusion, the article by Seok *et al.* (5) can be seen as part of a second generation of research into the neural correlates of mental health. This second generation of research embraces the multivariate brain bases of mental health by leveraging data-driven approaches and carefully evaluating analytical flexibility. The second generation of research also understands realistic brain-symptom effect sizes and uses larger samples and rigorous tests in held-out data to improve replicability, generalizability, and clinical translatability of insights.
